# Diverse Macrophages Constituted the Glioma Microenvironment and Influenced by PTEN Status

**DOI:** 10.3389/fimmu.2022.841404

**Published:** 2022-02-21

**Authors:** Fengqi Zhou, Qinyu Shi, Xiao Fan, Ruilei Yu, Zhiqiang Wu, Binbin Wang, Wei Tian, Tianfu Yu, Minhong Pan, Yongping You, Yingyi Wang

**Affiliations:** ^1^ Department of Neurosurgery, The First Affiliated Hospital of Nanjing Medical University, Nanjing, China; ^2^ Department of Radiology, The First Affiliated Hospital of Nanjing Medical University, Nanjing, China; ^3^ Department of Neurosurgery, The Affiliated Hospital of Nanjing University Medical School, Nanjing, China; ^4^ Department of Pathology, The First Affiliated Hospital of Nanjing Medical University, Nanjing, China

**Keywords:** glioma, immune microenvironment, macrophages, PTEN, polarization

## Abstract

The glioma immune microenvironment (GIM), consisting of glioma cells, stromal cells, and immune cells, accelerates the initiation, development, immune evasion, chemoresistance, and radioresistance of glioblastoma (GBM), whereas the immunosuppressive mechanisms of GBM have not been thoroughly elucidated to date. The glioma data downloaded from The Cancer Genome Atlas (TCGA) and the Chinese Glioma Genome Atlas (CGGA) databases were used to evaluate the composition of tumor-infiltrating immune cells (TICs) by the CIBERSORT algorithm. RNA-seq datasets from the TCGA and CGGA were used to analyze the relationship between immune scores with patients’ characteristics and TICs, which showed higher ratios of tumor-inhibiting/tumor-promoting signatures (M2/M1 macrophages) along with higher immune scores. The distribution of TICs among different glioma patients and the correlation with hazard ratio (HR) analysis suggested that M2 macrophages were abundant in malignant gliomas and indicated an unfavorable prognosis. We further analyzed TCGA cases with available mutation and copy-number alteration information, which showed that the status of PTEN could influence the immune microenvironment of glioma patients. Tissue microarrays of 39 GBM patients were carried out to confirm the clinical significance of PTEN and macrophage markers. We found that the high expression of PTEN was associated with a more extended survival period of glioma patients, positively correlated with M2 macrophages and negatively with M1 macrophages. Transwell and flow cytometry analyses demonstrated that PTEN status could prevent M1 to M2 polarization and M2 macrophage recruitment of gliomas *in vitro.* The newly discovered immunoregulatory activity of PTEN opens innovative avenues for investigations relevant to counteracting cancer development and progression.

## Introduction

Gliomas comprise nearly four-fifths of all common malignant central nervous system tumors, which include low-grade glioma (LGG) and glioblastoma (GBM) (1). LGG, according to the criteria established by the World Health Organization (WHO), defined as a lower grade (I–III), has a more favorable prognosis. Conversely, GBM classified as the highest grade (IV) has a more unfavorable prognosis (2, 3). Moreover, GBM is one of the most highly immunosuppressive and lethal tumors. Although standard therapies had improved, concluding surgical resection, radiotherapy, systemic therapy (chemotherapy, targeted therapy) (4), and supportive care, the median survival of GBM patients remains less than 16 months (5, 6), and treatment remains palliative.

Given that multiple components composed the glioma microenvironment, including blood vessels, parenchyma cells, extracellular matrix, soluble factors, and infiltrating immune cells (7, 8), tumor-associated macrophages (TAMs) are enriched in GBMs, serving as an essential element of the tumor microenvironment in GBM and playing crucial roles in supporting tumor growth (9, 10), suggesting that targeting TAMs may improve tumor outcome. It is well known that TAMs include two key populations: M2 macrophages serving as tumor supporters and M1 macrophages serving as tumor suppressors (11). In the tumor microenvironment, M2 macrophages play immune-suppressive roles *via* promoting tumor development (12). What is more, the majority of TAMs in GBMs have M2-like properties (13), which is verified in several studies that M2-polarized TAMs support the malignant growth of GBM (14–16). Although M2 macrophages have a remarkable effect on the development of GBMs, the mechanisms underlying the program and maintenance of M2 macrophages in the glioma microenvironment remain unknown.

Phosphatase and tensin homolog (PTEN) is an omnipresently expressed tumor suppressor, commonly inactivated in sporadic human cancers. In many tumors such as brain, prostate, and bladder, PTEN frequently showed genomic deletion (17, 18). Shortly after that, PTEN mutations were also found to increase the risks of cancer predisposition and neurological symptoms (19). PTEN is a central negative regulator of the phosphoinositide 3-kinase (PI3K)-Akt-mechanistic target of rapamycin (mTOR) signaling pathway, which plays a significant role in ruling a wide range of essential cellular processes such as cell proliferation, apoptosis, survival, and metabolism (20, 21).

In this study, we addressed glioma classification by identifying specific immune types based on The Cancer Genome Atlas (TCGA) and the Chinese Glioma Genome Atlas (CGGA) database. Glioma immune scores may provide novel insights into the glioma microenvironment, systematically investigating clinical, genomic, and biological conditions. M2 macrophages, existing more in the higher immune score patients, were influenced by PTEN status.

## Material and Methods

### Human Glioma Samples

Thirty-nine surgical resection specimens of glioma patients were obtained from the First Affiliated Hospital of Nanjing Medical University (Nanjing, China). All the specimens’ histological features were identified by pathologists following the WHO criteria. This study protocol was approved by the Research Ethics Committee of Nanjing Medical University (Nanjing, Jiangsu, China).

### Glioma Patient Databases

Databases from patients were downloaded from the TCGA database (*n* = 688) (http://cancergenome.nih.gov/) and CGGA database (*n* = 693) (http://www.cgga.org.cn). DNA copy number and mutation profiles were obtained from the TCGA website (http://cancergenome.nih.gov/).

### Western Blot Analysis

Western blot analysis was performed as described previously (22). Immunoblot analysis used the following primary antibodies: anti-PTEN (#9188), Akt (#4685), phospho-Akt (#4060), CD163 (#93498), CD68 (#97778), CD206 (#24595), iNOS (#13120), and α-tubulin (#2144) were purchased from Cell Signaling Technology (MA, USA).

### Flow Cytometry

Cells were incubated with fluorescence-conjugated antibodies for 30 min at 4°C, after being collected and washed. Cells were fixed and permeabilized with a fixation/permeabilization solution kit (BD Cytofix/Cytoperm) and 1% paraformaldehyde (PFA) for facilitating intracellular staining. The results were analyzed using the FlowJo 10.7 software program. This analysis used the following primary antibodies: Iba1 (#17198) and anti-rabbit IgG (H+L), F(ab′)2 fragment (PE conjugate) (#79408) were purchased from Cell Signaling Technology (MA, USA), and CD163-APC (130-100-612) was purchased from Miltenyi Biotec (Germany).

### Polarization of Macrophages

For differentiating into an intermediate stage M0, THP-1 cells were stimulated by phorbol 12-myristate 13-acetate (PMA). After that, IFN-γ and LPS were used to stimulate the polarization from M0 to M1 macrophages and then IL-4 and IL-13 for the polarization from M0 into M2 macrophages.

### Cell Lines, Incubation, and Stable Cell Establishment

Three human GBM cell lines (U251, LN229, and U87) and the human monocyte cell line THP-1 cells were purchased from the Chinese Cell Repository (Shanghai, China). LN229 cells were infected with lentivirus containing PTEN-specific shRNA (shPTEN) or non-targeting control shRNA (shCONT). For establishing stably expressing shPTEN or shCONT, transfected LN229 cells were selected with additional 5 mg/ml puromycin for 3 to 4 weeks. The targeting sequence for PTEN-shRNA was 5′-GCTAGAACTTATCAAACCCTT-3′.

### Macrophage Migration Assay

After differentiating THP-1 cells into M2 macrophages *in vitro*, the macrophage migration assay was performed. In a 24-well plate, U87, U251, LN229^shCONT^, and LN229^shPTEN^ cells were placed on the bottom of the lower chamber serving as a chemoattractant. Meanwhile, M2 macrophages were added in the upper transwell inserts (New York, USA, Cat: 09717050). After being incubated at 37°C and 5% CO_2_ for 48 h, the remaining cells on the top of the membrane were wiped off with a cotton swab. Cells which migrated into the bottom of the membrane were fixed with 4% PFA and stained with crystal violet solution. After dipping into distilled water to remove excess staining, the results of migration were visualized under a microscope.

### M1 Macrophage and Glioma Cell Transwell Co-Culture Assay

Cell culture inserts (3.0 µm) (New York, USA, Cat: 353492) were used to perform this indirect co-culture assay. In the presence of PMA, U87, U251, LN229^shCONT^, and LN229^shPTEN^ cells were seeded into the bottom wells. Meanwhile, the upper inserts were added with M1-polarized THP-1 cells. Macrophages were then collected and labeled with M2 macrophage marker CD163 and pan-macrophage marker Iba1 to identify the phenotypic changes using flow cytometry.

### Estimation of TICs

After uploading the normalized gene expression data to the CIBERSORT web portal (https://cibersort.stanford.edu/), 100 permutations and the LM22 gene signature were used to determine the algorithm. The 22 immune cells mainly consisted of T cells, B cells, NK cells, and other myeloid subsets (23). TICs from tumor and normal samples were differentiated by principal component analysis (PCA). Cases with a CIBERSORT algorithm *p*-value <0.05 were filtered and selected for further analysis.

### Immunohistochemistry

All immunohistochemistry experiments were performed according to the methods described in our previous study (24). Histochemistry score analyses were conducted by using the IHC profiler through ImageJ.

### *In-Vivo* Tumorigenesis

Viable GBM cells were counted and engrafted intracranially into female Bagg albino (BALB)/c nude mice (4 weeks old). All the animal experiments were approved in accordance with the Animal Use Guidelines of the Chinese Ministry of Health (documentation 55,2001). Healthy female mice of Bagg albino (BALB)/c background, 4 weeks old, were randomly selected and used in this study for intracranial injection. Animals were maintained until neurological signs were apparent, at which point they were sacrificed. Brains were collected and fixed in 4% formaldehyde, cryopreserved in 30% sucrose, and then cryosectioned.

### Immunofluorescent Analysis

Tumor sections were fixed with 4% paraformaldehyde, washed with PBS, permeabilized with 0.5% (vol/vol) Triton X-100 (California, USA, 1610407), and blocked with 3% (wt/vol) BSA (Saint Louis, USA, A7906) in PBS. Primary antibodies [anti-Iba1 (Cell Signaling Technology, #17198) and anti-CD163 (Boston, MA, USA, ab156769)] were added to the sections and incubated overnight at 4°C. After three washes with cold PBS, samples were incubated with secondary antibodies [Alex 488- or 568-labeled anti-rabbit or anti-mouse (Thermo Fisher Scientific)] for 1 h at room temperature, counterstained with 4,6-dianmino-2-phenylindole (DAPI) (Cell Signaling, 4083, 1:5,000), and sealed with mounting medium (Sigma-Aldrich, F4680).

### Statistical Analysis

The differences in the variable groups were assessed by Student’s *t*-test and one-way analysis of variance (one-way ANOVA). SPSS 24.0 (IBM, Chicago, USA), R3.3.1 (https://www.r-project.org/), and GraphPad Prism 8.0 programs were used for statistical analyses. The levels of infiltrating stromal and immune cells and tumor purity in the tumor samples were calculated by “Estimation of Stromal and Immune cells in Malignant Tumors using Expression data” (ESTIMATE) (25). The Gene Tree View software was used to generate heatmaps in this study. Kaplan–Meier curves and the log-rank test were used to conduct survival analysis with GraphPad Prism 8 software. Statistical values of *p <*0.05 were considered to be significant.

## Results

### The Immune Cell Infiltration Landscape in Gliomas

**Figure 1:**
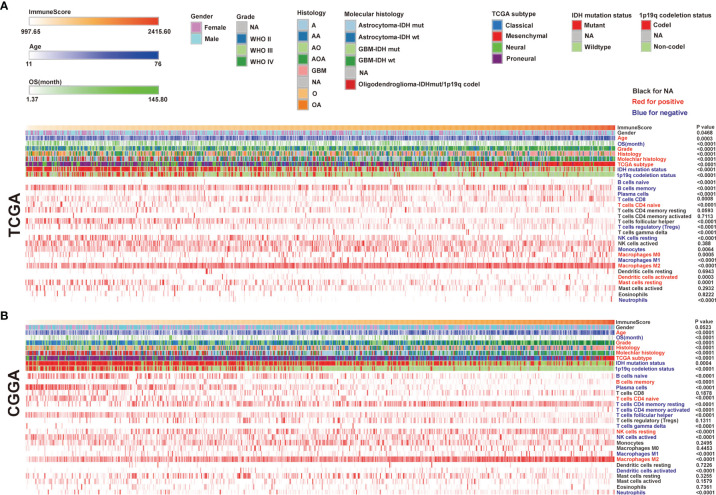
The landscape of clinical and molecular characteristics in association with immune scores. The Cancer Genome Atlas (TCGA) **(A)** and the Chinese Glioma Genome Atlas (CGGA) **(B)** sets were arranged to increase immune scores. The relationship between immune scores and patients’ characteristics was evaluated. Heatmaps of the enrichment levels of 22 immune cell types correlated with the immune scores in the TCGA and CGGA.

RNA-seq datasets used in this study were downloaded from the TCGA and CGGA. Gliomas from the two datasets were arranged in order of increasing immune scores. The distribution of immune scores was evaluated among different groups and associated with other factors. We found a positive correlation between glioma immune scores and clinical or molecular characteristics, including age, grade, and histology. Moreover, patients who got lower immune scores have better overall survival (OS) than those who got higher immune scores. The mesenchymal subtype gliomas display higher immune scores compared with the other three subtypes. Concerning genomic alterations, in glioma samples, the IDH mutation and 1p/19q codeletion indicated lower immune scores (**Figures 1A, B**). In addition, we evaluated the different enrichment levels of 22 immune cell types associated with the immune scores. Furthermore, the 22 tumor-infiltrating immune cell (TIC) cluster heatmaps are shown in **Figures 1A, B**. We noticed that B cells naive, B cells memory, plasma cells, T cells CD8, Tregs, NK cells resting, monocytes, macrophages M1, and neutrophils were negatively correlated with the immune score in the TCGA, while B cells naive, plasma cells, T cells CD4 (memory resting/activated), T cells (follicular helper/gamma delta), NK cells active, macrophages M1, dendritic cells activated, and neutrophils were negatively correlated with the immune score in the CGGA as well. According to the above findings, B cells naive, plasma cells, macrophages M1, and neutrophils were negatively correlated with the immune score in both the TCGA and the CGGA. We also observed that T cells (CD4 naive), macrophages M0 and M2, dendritic cells activated, and mast cells resting were positively correlated with the immune score in the TCGA, while B cells memory, T cells (CD4 naive), NK cells resting, and macrophages M2 were positively correlated with the immune score in the CGGA as well. It was quickly found that macrophages M2 and T cells (CD4 naive) were positively correlated with the immune scores in both the TCGA and the CGGA. Interestingly, we observed higher ratios of tumor-inhibiting/tumor-promoting signatures (M2/M1 macrophages) in higher immune scores than in immune scores–low gliomas.

### Different Distribution of TICs Among Glioma Grades and Subtypes

**Figure 2:**
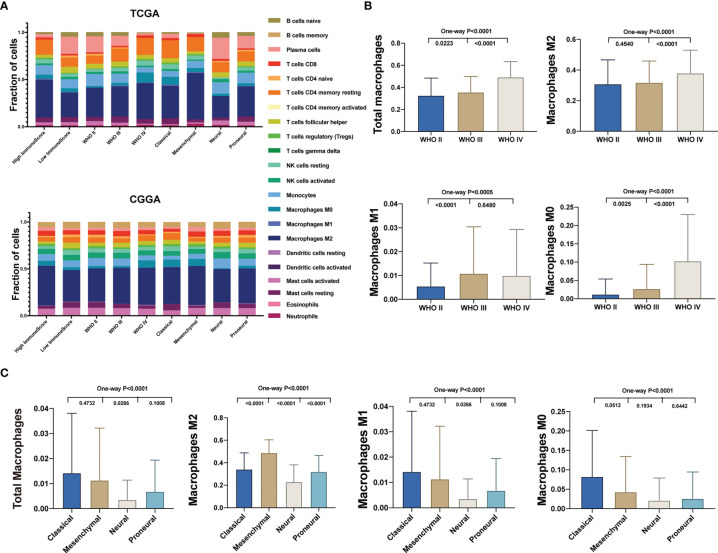
Different distribution of TICs among glioma subtypes. **(A)** The fractions of 22 immune type cells in different glioma patients’ characteristics in the TCGA (top) and CGGA (bottom). **(B)** Comparison of the total and different subtypes of macrophage expression between different WHO grade glioma specimens in the TCGA. **(C)** Comparison of the total and different subtypes of macrophage expression between TCGA subtypes in the TCGA.

Then we evaluated the distribution of the 22 TICs in different glioma groups, distinguished by immune scores, WHO grade, and TCGA subtypes using the TCGA (top) and CGGA datasets (bottom) (**Figure 2A**). In the TCGA, we found that the expression of tumor supporter M2 macrophages in the WHO IV group was the highest compared with the other two histology groups (WHO II and WHO III) and in the mesenchymal group compared with the other three subtypes (classical, neural, and proneural) (**Figures 2B, C**). In the CGGA, M2 macrophages’ expression was higher in WHO IV than in WHO II (**Supplementary Figure 1A**) and the lowest in the neural subtypes (**Supplementary Figure 1B**). Total macrophages and M0 macrophages existed more in WHO IV compared with WHO III or II, both in the TCGA and CGGA (**Figure 2B** and **Supplementary Figure 1A**). The expression of total macrophages has no statistical difference among the four subtypes, whereas M0 macrophages’ expression showed in the neural subtype was the lowest (**Supplementary Figure 1B**). As for the expression of M1 macrophages, there was no statistical difference among WHO IV, III, and II or the four subtypes in the CGGA, although it was the lowest in WHO II or the highest in the classical subtype in TCGA (**Figures 2B, C** and **Supplementary Figures 1A, B**). In conclusion, we found that more malignant gliomas generally had a higher expression of M2 macrophages.

### M2 Macrophages Indicated an Unfavorable Prognosis

**Figure 3:**
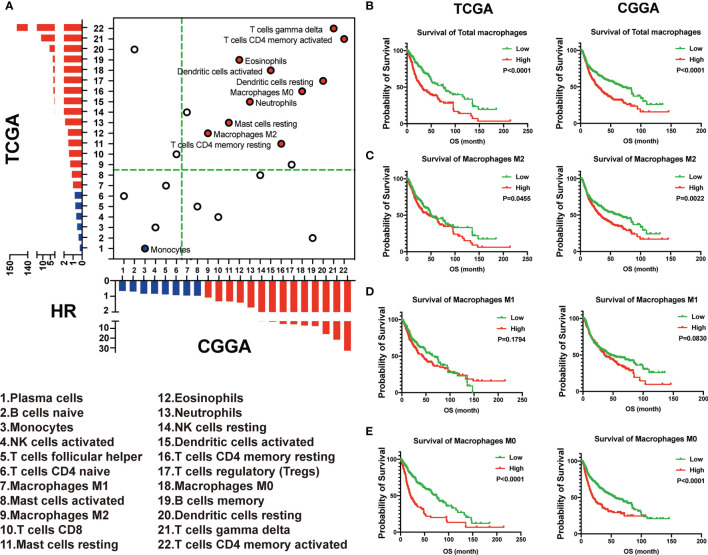
M2 macrophages indicated an unfavorable prognosis. **(A)** The 22 immune cell types correlated with hazard ratio (HR) in the TCGA and CGGA. **(B–E)** The total and different subtypes of macrophages were associated with the prognosis in glioma patients in the TCGA (left) and CGGA (right), based on the results of the Kaplan–Meier survival analysis.

The 22 immune cell types correlated with hazard ratio (HR) in the TCGA and CGGA were analyzed. We observed that monocytes were correlated with favorable outcomes both in the TCGA and CGGA. On the other hand, 10 of these 22 TICs were related to adverse outcomes, including M2 macrophages, which positively correlated with the immune score both in the TCGA and CGGA (**Figure 3A**). The dichotomization on median value was used to separate cases for depicting the survival curves. Parallel analyses were conducted in both the TCGA and CGGA datasets. We found that the high expression of total macrophages and M0 and M2 macrophages was associated with a shorter survival period in both the TCGA and CGGA datasets. On the contrary, there was no significantly different OS between higher and lower M1 macrophages in both the TCGA and CGGA datasets (**Figures 3B**–**E**). Taken together, these data suggested that M2 macrophages indicated an unfavorable prognosis.

Total macrophage enrichment is associated with distinct patterns of genomic alterations, and the glioma prognosis was correlated with the status of PTEN.

**Figure 4:**
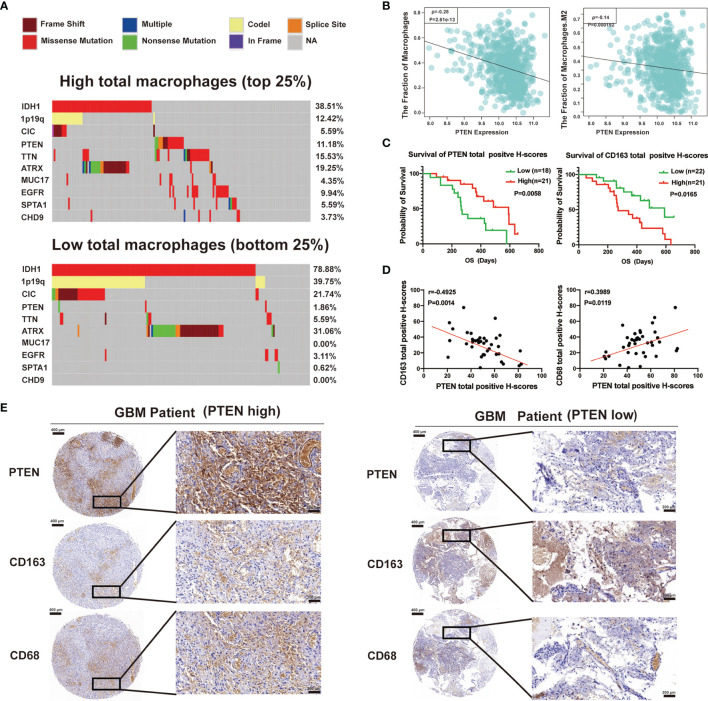
Total macrophage enrichment is associated with distinct patterns of genomic alterations, and the glioma prognosis was correlated with the status of phosphatase and tensin homolog (PTEN). **(A)** Differential somatic mutations were detected by comparing gliomas with high and low total macrophages. **(B)** The relationship of total macrophages (left) or M2 macrophages (right) with the expression of PTEN in the TCGA database. **(C)** Kaplan–Meier curves of overall survival for patients with high or low total positive H-scores of PTEN (left) and CD163 (right). **(D)** Spearman correlation analysis between PTEN and CD163 (left) or CD68 (right) protein levels in GBM specimens. **(E)** Immunohistochemical staining of PTEN, CD163, and CD68 in human GBM tissues. Representative images of immunohistochemical staining from the same tumor samples (GBM patient PTEN high, left; GBM patient PTEN low, right) are shown. Scale bar: 400 µm (left), 200 µm (right).

For uncovering the molecular mechanisms that influence tumor cell percentage within the glioma microenvironment, we analyzed TCGA cases with available mutation and copy-number alteration information. Based on the increasing total macrophages, cases were divided into four subgroups. Here, we showed the first quarter (low total macrophages) and fourth quarter (high total macrophages), frequent mutations in IDH1, 1p19q, PTEN, CIC, TTN, ATRX, and EGFR. Interestingly, PTEN mutations (including nonsense and missense mutations) almost happened in the high total macrophage group (**Figure 4A**). We also found that the expression of PTEN was negatively associated with the fraction of total macrophages and M2 macrophages (**Figure 4B**). To assess our findings’ clinical relevance, we have used tissue microarrays containing 39 GBM patients to carry out the immunohistochemical analysis of PTEN, CD68 (M1 macrophage marker), and CD163 (M2 macrophage marker). We further evaluated whether the expression of PTEN, CD163, and CD68 was associated with the prognosis of GBM patients. Kaplan–Meier survival analysis indicated that patients with low expression of PTEN exhibited much worse OS. On the contrary, increased expression of CD163 was associated with a more extended survival period (**Figure 4C**), and the expression of CD68 had no significant association with the OS of GBM patients (**Supplementary Figure 2A**). A highly significant positive correlation between PTEN and CD68 was found in these GBM specimens. In contrast, a negative correlation was found between PTEN and CD163 (**Figure 4D**). CD163 and CD68 expression levels also have an opposing correlation (**Supplementary Figure 2B**). Tumors with high total positive histochemistry scores (H-scores) (the summary of high positive, positive, and low positive H-scores) of PTEN expressed low CD163 and high CD68 levels (**Figures 4D, E**). Collectively, the status of PTEN was related not only to the polarization of macrophages in the glioma microenvironment but also to the prognosis of glioma patients.

PTEN impairs the macrophage polarization from M1 toward M2 and the recruitment of M2 macrophages.

**Figure 5:**
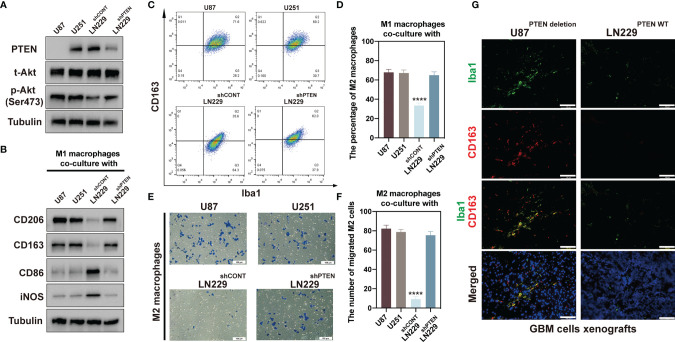
PTEN deletion or mutation glioma cells promoted M1 macrophage differentiation toward M2 macrophage. **(A)** Immunoblot analyses of PTEN, total Akt, and phosphor-Akt (Ser473) expression in cell lysates of U87, U251, LN229^shCONT^, and LN229^shPTEN^ cells. Tubulin served as the loading control. **(B)** Immunoblot analyses of CD206, CD163, CD86, and iNOS expression of macrophages after co-culture with U87, U251, LN229^shCONT^, and LN229^shPTEN^ cells. Tubulin served as the loading control. **(C)** Flow cytometric analysis for pan-macrophage marker Iba1 and M2 macrophage marker CD163 expression in the collected macrophages after co-culture with U87, U251, LN229^shCONT^, and LN229^shPTEN^ cells. **(D)** The percentage of M2 macrophages is summarized in the bar chart. **(E)** Representatives of M2 macrophage migration assays induced by U87, U251, LN229^shCONT^, and LN229^shPTEN^ cells. Scale bar: 100 µm. **(F)** Statistical analysis of the percentage of the migrated M2 macrophage cells. The data were calculated as means ± standard error of the mean (SEM) of six independent experiments (right). **(G)** Immunofluorescent analysis of the pan macrophage marker Iba1 (green) and M2 marker CD163 (red) in GBM xenografts derived from U87 cells or LN229 cells. Frozen sections of these GBM xenografts were immunostained with antibodies against Iba1 and CD163 and counterstained with DAPI (blue). Marked reductions of CD163 and Iba1 signals were detected in the LN229-derived tumors. Scale bars: 100 µm (****p < 0.0001).

To further examine the relationship between PTEN deletion/mutation and M2 macrophage infiltration *in vitro*, we chose U87 (PTEN deletion), U251 (PTEN mutation), and LN229 (PTEN wild type) cells for further study. Moreover, LN229 cells were transduced with a non-targeting control small hairpin RNA (shRNA) (shCONT) and shRNA targeting *PTEN* (shPTEN), respectively. We performed Western blot analyses of these glioma cell lines. The results revealed that PTEN was not expressed in U87 cells, and the PTEN expression was silenced with shPTEN in LN229^shPTEN^. The levels of phosphorylated Akt (Ser 473) were significantly lower in LN229^shCONT^ compared with the other cell lines (U87, U251, and LN229^shPTEN^) (**Figure 5A**). We established a co-culture system of glioma cells with M1 or M2 macrophages. After polarization of THP-1 cells into M2 macrophages, we subsequently collected M1 macrophages co-cultured with U87, U251, LN229^shCONT^, and LN229^shPTEN^ cells, respectively, for Western blot and flow cytometry analyses. Western blot analysis results showed that the expression levels of M2 macrophage markers CD206 and CD163 in these macrophages co-cultured with PTEN deletion, mutation, or silenced cells (U87, U251, and LN229^shPTEN^) were significantly higher compared with those co-cultured with PTEN wild-type glioma cells (LN229^shCONT^). On the contrary, the macrophages co-cultured with LN229^shCONT^ showed higher expression levels of M1 macrophage markers CD86 and iNOS (**Figure 5B**). The flow cytometry results revealed that the percentage of M2 macrophages (CD163^+^/Iba1^+^) was higher after being co-cultured with U87, U251, and LN229^shPTEN^ cells compared with LN229^shCONT^ cells (**Figure 5C**), and the statistical analysis of the percentage of M2 macrophages is shown in **Figure 5D**. To determine whether PTEN status could impact M2 macrophage migration, we subsequently co-cultured M2 macrophages, respectively, with U87, U251, LN229^shCONT^, and LN229^shPTEN^ cells after polarizing THP-1 cells into M2 macrophages. The results revealed that the migration of M2 macrophages was remarkably decreased when co-cultured with LN229^shCONT^ cells (PTEN wild type) (**Figure 5E**), and the statistical analysis of the percentage of migrated M2 macrophage cells is shown in **Figure 5F**. To address whether PTEN plays a role in TAM recruitment, we examined the effect of PTEN deletion or wild type on TAM density in GBM cell xenografts. We applied the M2-specific marker CD163 and the pan macrophage marker Iba1 to distinguish M2 TAMs in GBM cell-derived tumors. Frozen sections of these GBM xenografts derived from U87 (PETN deletion) cells or LN229 (PETN wild type) cells were immunostained with antibodies against Iba1 and CD163 and counterstained with DAPI (**Figure 5G**). As shown by the immunofluorescent analysis, marked reductions of CD163 and Iba1 signals were detected in the PTEN wild-type-derived tumors. These findings revealed that PTEN is an essential factor in preventing the polarization of M1 macrophages toward M2 macrophages and the migration of M2 macrophages *in vitro* and *in vivo*.

## Discussion

GBM is the most well-known primary brain malignancy in adults, characterized with extensively cellular heterogeneity and hierarchy. Diverse phenotypic tumor cells, prominent TAMs, other immune cells, and the extracellular matrix all together dispatch tumor progression and immune evasion (2, 26). The communication within those cells, including vascular cells, stromal cells, transformed cells, and infiltrating inflammatory cells in the tumor microenvironment, contributed to the poor prognosis of patients (10). Through the TCGA and CGGA databases, we evaluated the different enrichment levels of 22 immune cell types among glioma samples based on immune scores. We demonstrated that patients with lower immune scores had better OS. A potential explanation for this is that the progression and exacerbation of gliomas could be promoted by the inflammatory tumor microenvironment (27). Our results also confirmed that a macrophage-rich microenvironment could shape a mesenchymal glioma cell phenotype. Noticeably, most IDH1 and 1p/19q codeletion mutations occurred in the group with low immune scores, indicating that IDH mutations and 1p/19q codeletion negatively correlate with glioma immunity. These findings are consistent with the results of a previous study showing that IDH mutations were associated with low immune infiltration in gliomas (28, 29).

In recent years, several studies have investigated the immune landscape of glioma. For instance, Thorsson et al. revealed that the immunologically quiet subtype mainly was composed of LGG, containing the lowest level of lymphocyte infiltration (30).

Total macrophages-high included a significantly higher percentage of PTEN mutated than total macrophages-low, suggesting that PTEN mutations have a negative correlation with glioma immunity. To explore the impact of different PTEN statuses on the immunological classification of gliomas, glioma cells were separated into PTEN deletion, PTEN mutation, PTEN wild type, and PTEN wild type-shPTEN groups for *in-vitro* experiments. The subsequent experiments validated that the change or loss of PTEN wild-type status could promote tumor cells to reprogram macrophages from M1 to M2 and facilitate the migration of M2 macrophages.

PTEN status detection in glioma has tremendous potential to raise our understanding of this disease’s pathology and identify efficient therapeutic approaches to improve clinical outcomes for glioma patients. As the most commonly lost tumor suppressor in primary glioma, PTEN loss, which served as one of the few prognostic biomarkers, is reproducibly associated with unfavorable prognosis in glioma patients. In conclusion, our results showed that the change of PTEN status could influence the immune response to tumor progression and has a key role in predicting which patients will respond to promising immunotherapies.

## Data Availability Statement

The original contributions presented in the study are included in the article/Supplementary Material. Further inquiries can be directed to the corresponding author.

## Ethics Statement

The studies involving human participants were reviewed and approved by Research Ethics Committee of Nanjing Medical University (Nanjing, Jiangsu, China). The patients/participants provided their written informed consent to participate in this study.

## Author Contributions

YW, YY, and MP conceived and designed the experiments. FZ performed all the *in-vitro* experiments with the assistance of XF, BW, and RY. QS performed all the *in-vivo* experiments with the assistance of ZW, WT, and TY. FZ wrote the manuscript. All authors reviewed the paper. All authors contributed to the article and approved the submitted version.

## Funding

This work was supported by grants from the National Natural Science Foundation of China (81772682) and the Priority Academic Program Development of Jiangsu Higher Education Institutions (PAPD).

## Conflict of Interest

The authors declare that the research was conducted in the absence of any commercial or financial relationships that could be construed as a potential conflict of interest.

## Publisher’s Note

All claims expressed in this article are solely those of the authors and do not necessarily represent those of their affiliated organizations, or those of the publisher, the editors and the reviewers. Any product that may be evaluated in this article, or claim that may be made by its manufacturer, is not guaranteed or endorsed by the publisher.
