# Supplementary material for: Diverse Macrophages Constituted the Glioma Microenvironment and Influenced by PTEN Status
Source: Front Immunol. 2022 Feb 21;13:841404. doi: 10.3389/fimmu.2022.841404 (PMC8899089; doi:10.3389/fimmu.2022.841404)
Supplement: Supplementary file 1 [file DataSheet_1.docx]

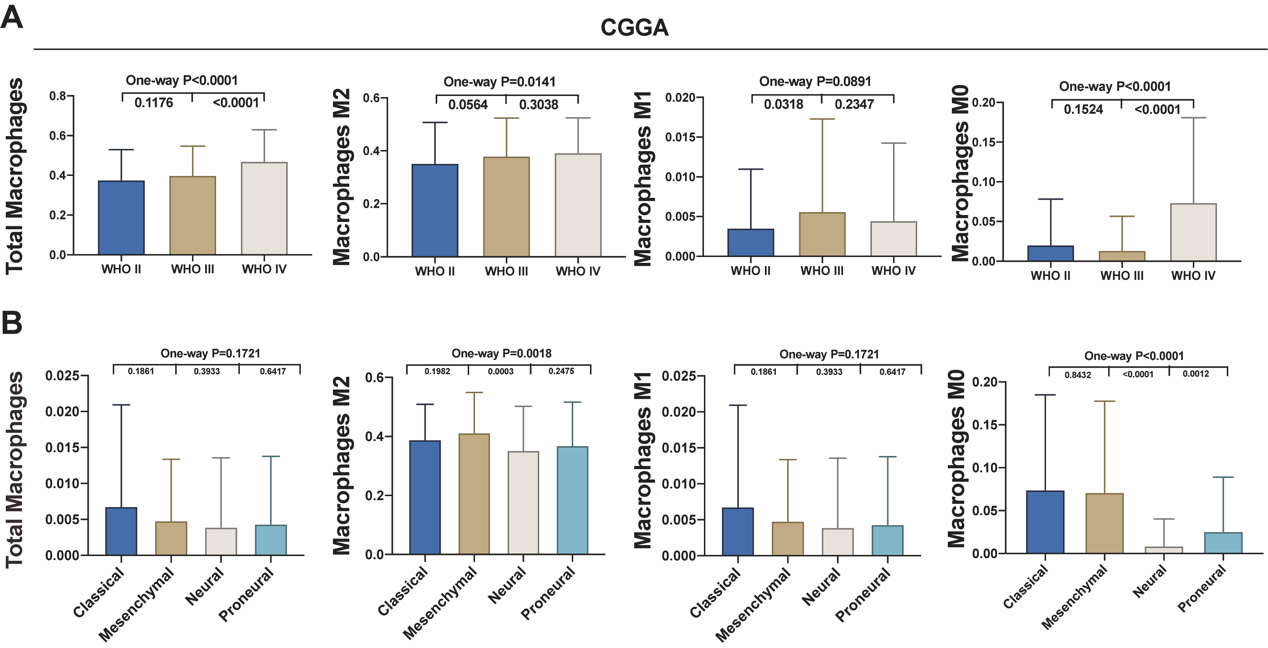


Supplementary Figure 1

(A) Comparison of total and different subtypes macrophages expression between different WHO grades of glioma specimens in the CGGA. (B) Comparison of total and different subtypes macrophages expression between different TCGA subtypes of glioma specimens in the CGGA.


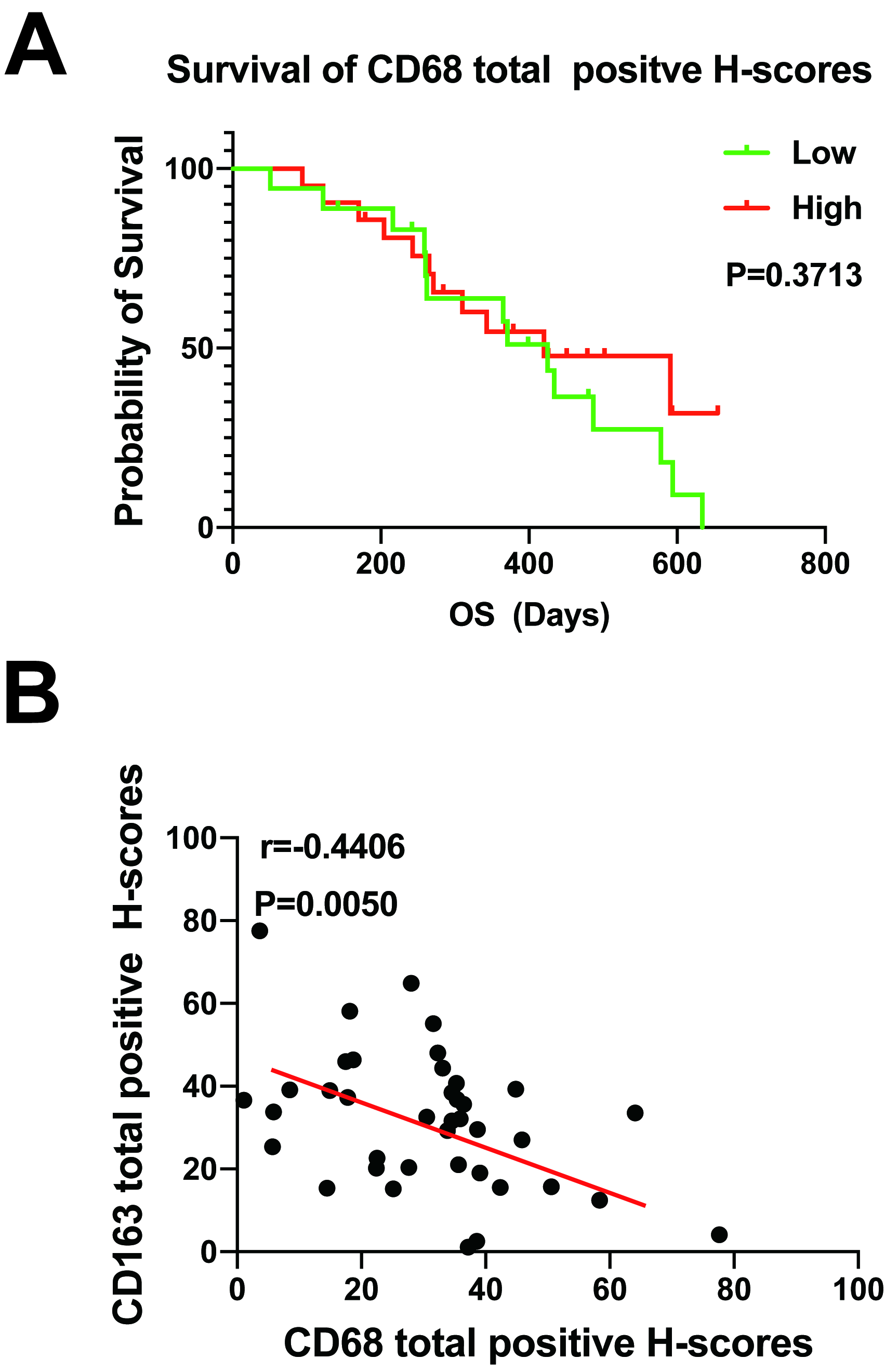


Supplementary Figure 2

(A) Kaplan–Meier curves of overall survival of for patients with high or low total positive H-scores of CD68. (B) Spearman correlation analysis between CD68 and CD163.
